# Refining Insect Pest Monitoring: How Crop and Pest Identity Influence the Choice of Sampling Unit and Technique

**DOI:** 10.1007/s13744-026-01401-x

**Published:** 2026-05-21

**Authors:** Adriano Cirino Tomaz, Francisco Sérgio Neres da Silva, Gerson Adriano Silva, Katiuchia Pereira Takeuchi

**Affiliations:** 1 Faculdade de Agronomia E Zootecnia, Universidade Federal de Mato Grosso (UFMT), Cuiabá, Brazil; 2 Faculdade de Agronomia e Zootecnia, Universidade Federal de Mato Grosso (UFMT), Cuiabá, Brazil; 3 Universidade Estadual Norte Fluminense Darcy Ribeiro, Rio de Janeiro, Brazil; 4 Faculdade de Nutrição, Universidade Federal de Mato Grosso (UFMT), Cuiabá, Brazil

**Keywords:** Integrated pest management, Insect sampling, Direct counting, Tray beating, Whitefly, Aphid

## Abstract

This study aimed to determine the most suitable sampling unit (apical, middle, or basal leaf strata) and sampling technique (direct counting or tray beating) for sap-sucking pest groups (aphids, thrips, lace bugs, and whiteflies) across different crops in an agroforestry system (AFS). The study was conducted with kale, okra, tomato, and scarlet eggplant over three cropping seasons (one in 2023 and two in 2024). The main pest species were *Lipaphis erysimi* on kale and *Aphis gossypii* on okra (aphids); *Corythaica passiflorae* on scarlet eggplant and *Gargaphia lunulata* on okra (lace bugs); *Bemisia tabaci* on kale and tomato (whiteflies); and *Frankliniella schultzei* on tomato and *Thrips* spp. on okra (thrips). A generalized linear mixed model (GLMM) with negative binomial distribution was used to evaluate the effects of sampling technique, sampling unit, and crop on pest density. Optimal sampling combinations were defined based on higher mean densities and lower coefficients of variation (CV). Direct counting was the most effective technique for aphids, whiteflies, and lace bugs, whereas tray beating was more efficient for thrips. However, the optimal sampling unit varied strongly according to crop–pest interactions. These results indicate that, although sampling techniques can be partially standardized by pest group, the selection of the sampling unit must consider crop-specific interactions. This approach improves sampling efficiency and supports decision-making in Integrated Pest Management programs in diversified agroecosystems such as AFS.

## Introduction

Sap-sucking insects are a major group of pests affecting horticultural crops, including aphids (Hemiptera: Aphididae), whiteflies (Hemiptera: Aleyrodidae), lace bugs (Hemiptera: Tingidae), and thrips (Thysanoptera: Thripidae). Both nymphs and adults colonize young shoots, inflorescences, and leaves, feeding continuously. Damage results from sap extraction, toxin injection, virus transmission, and sooty mold development**.** This feeding activity induces chlorosis that may progress to necrosis, thereby reducing the photosynthetic area, plant productivity, and, under severe infestations, leading to plant death (Araújo et al. 2019; Carmo et al. 2021; Lima et al. 2017; Silva et al. 2024). Thrips, although differing in feeding behavior, are also important pests in tropical and subtropical regions. Both adults and nymphs rasp leaf tissues and extract cell contents, in addition to injecting toxins and transmitting viruses, which may result in yield losses (Bacci et al. 2008; Pinto et al. 2017).

Integrated Pest Management (IPM) is widely considered the most suitable strategy for controlling these pests in several crops (Zhou et al. 2024). IPM integrates compatible techniques to maintain pest populations below levels that cause economic damage. Within this framework, a species is considered a pest when its population exceeds the economic damage threshold (EDT)**,** defined as the density at which economic loss equals the cost of control (Zhou et al. 2024). Accurate pest sampling is a fundamental component of IPM, as it enables reliable estimation of pest density, the key variable for decision-making (Silva et al. 2025). A conventional sampling plan comprises three elements: sampling unit, sampling technique, and sample size.

The sampling unit is the plant structure where the target population is observed. It varies according to pest species and host plant and typically corresponds to the most affected organ, such as leaves, stems, flowers, or fruits (Araújo et al. 2019; Carmo et al. 2021). Proper selection of the sampling unit is essential to ensure representative and reliable data. Sampling techniques are defined after selecting the sampling unit and aim to accurately estimate insect densities. The most commonly used methods in IPM include direct counting and tray beating. Direct counting consists of carefully turning the leaf, usually by the petiole, to count insects on the abaxial surface while minimizing the escape of mobile species such as whiteflies. Tray beating consists of shaking plant parts over a white tray to dislodge insects for counting (Bacci et al. 2008; Macedo et al. 2019).

The selection of sampling units and techniques is based on criteria that balance accuracy and feasibility. Frequency of occurrence prioritizes plant structures that are consistently present. Relative variance (RV) is used as a measure of precision, and representativeness is assessed by the correlation between relative and absolute densities (Araújo et al. 2019; Carmo et al. 2021; Macedo et al. 2019). Economic factors, including sampling time, labor, and material costs, are also considered and integrated into indicators such as sampling cost and economic precision (Gusmão et al. 2005; Carmo et al. 2021; Lima et al. 2017). Recent advances in pest sampling have focused on improving precision while reducing sampling effort and operational costs, thereby enhancing decision-making efficiency in pest management programs.

Several studies have developed sampling methods for specific pest–crop combinations. For example, for *Bemisia tabaci*, recommended methods vary among crops: tray beating on an apical leaf in tomato (*Solanum lycopersicum*) (Gusmão et al. 2005), direct counting on the sixth most apical leaf in watermelon (*Citrullus lanatus*) and melon (*Cucumis melo*) (Lima et al. 2017; Macedo et al. 2019).

Similarly, for thrips, recommended methods vary by species and crop. For *Thrips palmi* in cucumber, tray beating on apical leaves is recommended, whereas for *Frankliniella schultzei*, direct counting is more effective (Bacci et al. 2008). In watermelon, direct counting is also recommended for *F. schultzei* (Pinto et al. 2017), whereas in tomato, tray beating on apical leaves is more efficient (Araújo et al. 2020).

For aphids, similar variation occurs. For *Aphis gossypii* in cotton (*Gossypium hirsutum*), direct counting on apical leaves is recommended (Araújo et al. 2019), whereas for *Myzus persicae* in bell pepper (*Capsicum annuum*), tray beating on the third or fifth apical leaf is more appropriate (Carmo et al. 2021).

These examples demonstrate that sampling methods depend strongly on the pest–crop combination and are generally species- and crop-specific. Such variation may arise from differences in methodology, insect behavior, spatial distribution, and plant architecture (Silva et al. 2025). Although expected, this variability limits standardization, particularly in diversified systems where multiple crops and pest species coexist.

Agroecological agroforestry systems (AFSs) have emerged as sustainable alternatives, characterized by high biological and structural diversity (Santos et al. 2021). However, crop and pest diversity limits the applicability of species-specific protocols for pest sampling in IPM of AFS**.** Therefore, this study aimed to determine the most suitable sampling method, considering both sampling techniques and sampling units, for different pest groups across multiple crops in an agroforestry system.

## Material and methods

### Climatic characteristics of the region

The research was conducted at the Experimental Farm of the Federal University of Mato Grosso, located in Santo Antônio do Leverger, Mato Grosso State, Brazil (15°51′01.7"S, 56°04′14.5"W), in a transition zone between the Pantanal and Cerrado biomes. The study was carried out in a reference agroforestry system (AFS) representative of the “Baixada Cuiabana” region.

The region has a well-defined rainy season (October to March) and dry season (April to September), with average monthly temperatures ranging from 22 °C to 27°C. Maximum temperatures reached 33–34°C in October and September, respectively, while minimum temperatures dropped to 18–19°C in July and June (Climatempo 2025).

### Development of the agroforestry system

The AFS measured 24 × 24 m, totaling 576 m^2^. Its implementation began in October 2022 with soil preparation and correction using lime and rock phosphate, based on soil analysis. Fifteen days after correction, perennial species were established by plant seedings in two rows, spaced 12 m apart, with 6 m between plants within rows: acerola (*Malpighia emarginata*), guava (*Psidium guajava*), cashew (*Anacardium occidentale*), and lemon (*Citrus limon*). Subsequently, additional crops were intercropped within the rows between the perennial crops, including papaya (*Carica papaya*), passion fruit (*Passiflora edulis*), banana (*Musa* spp.), pineapple (*Ananas comosus*), and cassava (*Manihot esculenta*).

The two rows of perennial species were established in an east–west orientation to optimize solar radiation interception throughout the day.

The vegetable crops evaluated were kale (*Brassica oleracea* var. butter), scarlet eggplant (*Solanum gilo* var. light green Tinguá), tomato (*Solanum lycopersicum* var. Italian), and okra (*Abelmoschus esculentus*). These crops represent distinct botanical families and are commonly cultivated in the Cuiabá lowland region. They were cultivated simultaneously within the same area, occupying the space between perennial rows.

Each vegetable crop was grown in two rows (20 m long), with 1.0 m spacing between rows and 0.5–1.0 m between plants within rows, depending on the crop. The distance between perennial rows and vegetable rows was 2.0 m.

**Fig. 1:**
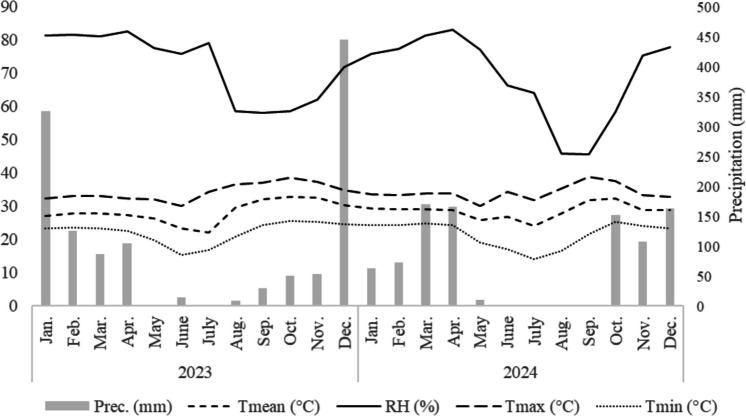
Monthly averages of climatic parameters in the municipality of Santo Antônio in 2023 and 2024. Prec. = Precipitation; Tmean = Mean temperature; RH = Relative humidity; Tmax = Maximum temperature; Tmin = Minimum temperature

Considering that most vegetables are negatively affected by excessive heat and rainfall, cultivation was scheduled from March to September, avoiding the rainy season and periods of high temperatures (Fig. 1). During the rainy season, maize was cultivated in the interrows to reflect regional agricultural practices and implement crop rotation.

### Crop management and experimental periods

Maize was cultivated in the interrows from January to March 2023. In April, maize residues were incorporated using a rotary tiller, followed by bed formation and installation of a drip irrigation system.

Seedlings of vegetable crops were produced in plastic trays during the first half of May and maintained in a greenhouse under micro-sprinkler irrigation. Transplanting occurred in the second half of May, using seedlings aged 15–30 days after germination, according to species-specific recommendations.

Fertilization was performed using cattle manure at approximately 300 g per plant. Throughout the crop cycle, agroecological practices were adopted, including manual weeding, mulching (dry leaf cover), and staking for tomato plants.

Pest population density was evaluated weekly from August 11 to September 8, 2023. Harvests were conducted according to crop recommendations: tomato and kale in the third week of September, and okra and scarlet eggplant in the fourth week.

From November 2023 to January 2024, maize was again cultivated in the interrows. In February, residues were incorporated, and beds and irrigation were re-established. Two cropping cycles were evaluated in 2024 (autumn and winter) to expand the temporal assessment of pest populations.

In the first cycle (2024), seedlings were produced in early February and transplanted in the second half of the month. Pest Evaluations were conducted weekly from April 12 to May 10, with harvests occurring in mid to late May.

In the second cycle, seedlings were produced in late May and transplanted shortly after the first harvest, in different beds to avoid reuse. Pest evaluations were conducted weekly from August 30 to September 30, 2024. Harvests followed crop-specific recommendations, with tomato and kale harvested in early September, and okra and scarlet eggplant in late September.

Pest evaluations were initiated when crops reached their productive stages—advanced vegetative stage in kale and reproductive stage in okra, tomato, and scarlet eggplant—approximately 80–130 days after transplanting. In all crop cycles, assessments were conducted weekly for four weeks.

### Insects

During crops development, the plants were regularly monitored for the presence of insects and plant injury symptoms. Insects observed feeding on plants were collected and taken to the Ecological Pest Management Laboratory (Agroecology Sector) for identification using a Novacom® digital microscope at 1000 × magnification.

Species identification was performed with the assistance of experienced entomologists, based on taxonomic keys (Gallo et al. 2002), and confirmed by comparison with images from online databases, technical documents, and specialized sources focused on insect morphology and plant injury characteristics.

Only insect species with consistent population occurrence in each crop were considered for the evaluation of sampling methods. The main pest species identified with significant infestation levels in each pest group were: Aphids *Lipaphis erysimi* (Hemiptera: Aphididae) on kale and *Aphis gossypii* (Hemiptera: Aphididae) on okra; Lace bugs *Corythaica passiflorae* (Hemiptera: Tingidae) on scarlet eggplant and *Gargaphia lunulata* (Hemiptera: Tingidae) on okra; whitefly *Bemisia tabaci* (Hemiptera: Aleyrodidae) on tomato and kale; and thrips *Frankliniella schultzei* (Thysanoptera: Thripidae) on tomato and *Thrips* spp. (Thysanoptera: Thripidae) on okra. Therefore, each pest group was assessed across two crop systems, allowing comparison of sampling performance under different host plant conditions.

### Determination of the sampling unit and technique

The sampling design consisted of six treatments, defined by the factorial combination of two sampling techniques (tray beating and direct counting) and three sampling unit (leaf of apical, middle and basal thirds).

The sampling techniques evaluated were tray beating and direct visual counting. In the tray beating method, a white tray (39 × 29 × 7 cm) was placed beneath a fully expanded leaf which was quickly shaken to dislodge insects onto the tray for counting. In the direct counting method, the leaf was carefully turned by the petiole to minimize insect disturbance, and all individuals present on the abaxial surface were counted (Araújo et al. 2019; Pinto et al. 2017).

At each evaluation date, ten plants per crop were randomly selected, with five plants assigned to each sampling technique. Each technique was applied to a different set of plants to ensure independence between methods. Within each plant, the sampling unit consisted of one fully expanded leaf from each of the three vertical strata (apical, middle and basal).

In each sampling event, both nymphs and adults of aphids, thrips, and lace bugs were counted either on the tray or directly on the leaves (Araújo et al. 2019; Pinto et al. 2017), whereas for whiteflies, only adults were considered (Lima et al. 2017).

### Data analysis

A generalized linear mixed model (GLMM) was fitted to evaluate the effects of sampling technique, sampling unit, and crop on pest density for each pest group. The model assumed a negative binomial distribution (nbinom2) with a log link function, which is appropriate for overdispersed count data.

The fixed effects included sampling technique (tray beating and direct counting), sampling unit (leaves from the apical, middle, and basal thirds), and crop (kale, scarlet eggplant, okra, and tomato), as well as all possible interactions among these factors. Random intercepts were included for plant identity and sampling date to account for repeated measurements and temporal variation.

Model significance was assessed using Type II Wald chi-square tests. Estimated marginal means (EMMs) were obtained and back-transformed to the response scale to generate biologically interpretable estimates of pest density. For each treatment combination, mean values and standard errors (SE) were calculated, and relative variability was expressed as the coefficient of variation (CV), computed as CV = (SE/mean) × 100**.**

Post hoc comparisons were performed using the emmeans package, and treatment performance was interpreted based on both mean estimates and coefficient of variation (CV), considering that lower variability indicates greater sampling reliability. All statistical analyses were performed using R software (R Core Team, 2025).

## Results

The generalized linear mixed models showed that the effects of sampling technique, sampling unit and crop varied among pest groups, with distinct interaction patterns (Table 1). The analysis of mean densities and coefficients of variation (CV)(Table 2. allowed the identification of optimal sampling combinations (Table 3), considering both detection sensitivity and sampling precision.

**Table 1:** Effects of factores on the density of several pest in different crops (GLMM, Wald χ2)

Pest	Effect	χ^2^ (Wald)	df	*p*-value	Significance
Aphids	Technique	157.91	1	< 0.0001	***
	Sampling unit	4.72	2	0.0942	ns
	Crop	15.89	1	< 0.0001	***
	Technique × Sampling unit	3.81	2	0.1488	ns
	Technique × Crop	2.98	1	0.0845	ns
	Sampling unit × Crop	32.44	2	< 0.0001	***
	Technique × Sampling unit × Crop	0.24	2	0.8874	ns
Thrips	Technique	52.3	1	< 0.0001	***
	Sampling unit	16.9	2	0.0002	***
	Crop	11.83	1	0.0006	***
	Technique × Sampling unit	16.84	2	0.0002	***
	Technique × Crop	2.38	1	0.1226	ns
	Sampling unit × Crop	4.67	2	0.0967	ns
	Technique × Sampling unit × Crop	1.69	2	0.4295	ns
Whitefly	Technique	18.57	1	< 0.0001	***
	Sampling unit	73.06	2	< 0.0001	***
	Crop	79.84	1	< 0.0001	***
	Technique × Sampling unit	11.38	2	0.0034	**
	Technique × Crop	1.5	1	0.2209	ns
	Sampling unit × Crop	53.13	2	< 0.0001	***
	Technique × Sampling unit × Crop	0.37	2	0.8329	ns
Lace bug	Technique	179.43	1	< 0.0001	***
	Sampling unit	28.65	2	< 0.0001	***
	Crop	52.29	1	< 0.0001	***
	Technique × Sampling unit	8.59	2	0.0136	*
	Technique × Crop	97.64	1	< 0.0001	***
	Sampling unit × Crop	20.99	2	< 0.0001	***
	Technique × Sampling unit × Crop	7.46	2	0.024	*

**Table 2:** Mean density (mean ± SE) and coefficient of variation (CV, %) of sap-sucking pests as affected by sampling technique and plant stratum across different crops in an agroforestry system

Crop	Technique	Sampling unit	Aphid		Thrips		Whitefly		Lace bug	
			Mean ± SE	CV (%)	Mean ± SE	CV (%)	Mean ± SE	CV (%)	Mean ± SE	CV (%)
Kale	Tray	Apical	0.16 ± 0.12	73.3	—	—	7.16 ± 2.89	40.4	—	—
		Basal	0.97 ± 0.67	69.7	—	—	11.26 ± 4.53	40.2	—	—
		Middle	0.35 ± 0.24	69.6	—	—	8.94 ± 3.60	40.3	—	—
	Counting	Apical	1.38 ± 0.98	70.9	—	—	15.92 ± 6.49	40.8	—	—
		Basal	16.28 ± 10.87	66.8	—	—	12.99 ± 5.28	40.6	—	—
		Middle	9.09 ± 6.29	69.2	—	—	15.90 ± 6.49	40.8	—	—
Okra	Tray	Apical	0.12 ± 0.09	75.2	0.10 ± 0.06	57.9	—	—	0.09 ± 0.06	62.6
		Basal	0.04 ± 0.03	73.3	0.13 ± 0.08	58.2	—	—	0.26 ± 0.16	60.5
		Middle	0.04 ± 0.03	80.5	0.07 ± 0.04	60.9	—	—	0.24 ± 0.14	60.7
	Counting	Apical	2.85 ± 1.96	69	0.09 ± 0.06	59.9	—	—	0.06 ± 0.04	64.7
		Basal	1.14 ± 0.79	69.2	0.02 ± 0.01	72	—	—	0.43 ± 0.26	59.9
		Middle	2.61 ± 1.76	67.4	0.08 ± 0.05	61.7	—	—	0.25 ± 0.15	60.9
Tomato	Tray	Apical	—	—	0.24 ± 0.13	54.7	0.84 ± 0.35	41.2	—	—
		Basal	—	—	0.28 ± 0.15	54.7	4.51 ± 1.83	40.5	—	—
		Middle	—	—	0.39 ± 0.21	54.5	4.79 ± 1.94	40.5	—	—
	Counting	Apical	—	—	0.11 ± 0.06	55.4	1.65 ± 0.68	41	—	—
		Basal	—	—	0.05 ± 0.03	56.4	4.44 ± 1.81	40.7	—	—
		Middle	—	—	0.18 ± 0.10	55.4	6.04 ± 2.47	41	—	—
Scarlet egg plant	Tray	Apical	—	—	—	—	—	—	0.35 ± 0.22	64.1
		Basal	—	—	—	—	—	—	0.36 ± 0.23	63.3
		Middle	—	—	—	—	—	—	0.37 ± 0.24	63.5
	Counting	Apical	—	—	—	—	—	—	3.95 ± 2.50	63.4
		Basal	—	—	—	—	—	—	4.13 ± 2.59	62.7
		Middle	—	—	—	—	—	—	15.43 ± 9.61	62.3

**Table 3:** Optimal sampling combinations for sap-sucking pest groups based on higher mean densities and lower coefficients of variation (CV) across crops in an agroforestry system

Pest	Crop	Optimal Technique	Optimal sampling unit	Mean ± SE	CV (%)
Aphids	Kale	Counting	Basal	16.28 ± 10.87	66.8
	Okra	Counting	Middle	2.61 ± 1.76	67.4
Thrips	Okra	Tray	Apical	0.10 ± 0.06	57.9
	Tomato	Tray	Middle	0.39 ± 0.21	54.5
Whitefly	Kale	Counting	Apical	15.92 ± 6.49	40.8
	Tomato	Counting	Middle	6.04 ± 2.47	41
Lace bug	Okra	Counting	Basal	0.43 ± 0.26	59.9
	Scarlet eggplant	Counting	Middle	15.43 ± 9.61	62.3

### Aphids

For aphids, sampling technique had a strong effect on density (χ2 = 157.91, *p* < 0.0001), and crop was also significant (χ2 = 15.89, *p* < 0.0001), whereas plant stratum alone was not significant (p = 0.0942). The stratum × crop interaction was highly significant (χ2 = 32.44, *p* < 0.0001), indicating that vertical distribution patterns depend on the host crop, while no interactions involving technique were significant.

The optimal aphid sampling was achieved using direct counting. In kale, the basal stratum presented the highest density with moderate variability (16.28 ± 10.87; CV = 66.8%), followed by the middle stratum (9.09 ± 6.29; CV = 69.2%). In okra, the middle stratum using counting provided the optimal combination (2.61 ± 1.76; CV = 67.4%). These results confirm that optimal sampling units vary among crops, consistent with the significant stratum × crop interaction.

### Thrips

Thrips densities were significantly affected by technique (χ2 = 52.3, *p* < 0.0001), stratum (χ2 = 16.9, *p* = 0.0002), and crop (χ2 = 11.83, *p* = 0.0006). A significant technique × stratum interaction (χ2 = 16.84, *p* = 0.0002) indicated that sampling efficiency varied across sampling unit, whereas other interactions were not significant.

Densities were generally low and variability moderate across crops. The optimal sampling combinations were tray beating in the apical stratum of okra (0.10 ± 0.06; CV = 57.9%) and middle stratum of tomato (0.39 ± 0.21; CV = 54.5%), balancing sensitivity and precision.

### Whitefly

Whitefly densities were significantly affected by technique (χ2 = 18.57, *p* < 0.0001), stratum (χ2 = 73.06, *p* < 0.0001), and crop (χ2 = 79.84, *p* < 0.0001). Significant interactions were observed for technique × stratum (χ2 = 11.38, *p* = 0.0034) and stratum × crop (χ2 = 53.13, *p* < 0.0001), showing that both sampling efficiency and insect distribution depend on plant position and crop type.

The optimal sampling combinations used counting. In kale, the apical stratum had the highest density and good precision (15.92 ± 6.49; CV = 40.8%), whereas in tomato, the middle stratum provided 6.04 ± 2.47 (CV = 41.0%). These results highlight the importance of jointly considering mean density and variability.

### Lace bugs

For lace bugs, all main effects and interactions—including the three-way technique × stratum × crop interaction (χ2 = 7.46, *p* = 0.024) were significant, indicating strong interdependence among factors.

Densities were low in okra and markedly higher in scarlet eggplant. The optimal combinations were counting in the basal stratum of okra (0.43 ± 0.26; CV = 59.9%) and counting in the middle stratum of scarlet eggplant (15.43 ± 9.61; CV = 62.3%), reflecting crop- and stratum-specific aggregation patterns.

## Discussion

The results demonstrate that the efficiency of sampling methods for sap-sucking insects is strongly influenced by the interaction between sampling technique, plant stratum, and crop, reflecting differences in insect behavior, spatial distribution, and host plant characteristics. These findings reinforce that sampling strategies cannot be generalized across pest groups without considering ecological and biological factors. Most sap-sucking insects, such as aphids, thrips, and whiteflies, exhibit an aggregated spatial distribution (Araújo et al. 2019; Bacci et al. 2008; Macedo et al. 2019). This means that individuals tend to form colonies or “patches” within crops, which explains the high coefficients of variation observed (> 40%).

For aphids, the lack of a significant effect of sampling unit alone, combined with the strong sampling unit × crop interaction, indicates that their vertical distribution varies according to host plant and species behavior. *Aphis gossypii*, found on okra, is highly polyphagous and occurs across a wide range of host plants (Araújo et al. 2019). This species typically exploits plant regions with higher nutritional quality, particularly tissues with greater availability of water and nitrogen, such as young or actively growing parts of the plant. In contrast, *Lipaphis erysimi*, associated with kale, is a key pest of Brassicaceae crops (Kalita et al. 2016) and showed higher densities in basal leaves, possibly due to leaf architecture and microclimatic conditions that favor colony establishment. The consistent superiority of direct counting for the aphid group is expected, given that aphids are relatively sessile insects that remain attached to plant surfaces. However, the optimal sampling unit depends on crop–species interactions.

For thrips, the significant interaction between sampling technique and plant stratum indicates that their detection is influenced by both insect behavior and plant structure. *Frankliniella schultzei*, identified in tomato, is a major pest of this crop in tropical regions (Araújo et al. 2020). The thrips associated with okra were identified only at the genus level (*Thrips* spp.), although the most common species in Brazil include *Thrips palmi* and *Thrips tabaci*, both polyphagous (Monteiro et al. 2001). *T. tabaci* primarily attacks Liliaceae and cotton, whereas *T. palmi* is commonly reported in cucurbits and solanaceous crops. In the present study, thrips densities were relatively low, with little variation among plant strata, suggesting a more dispersed distribution within the plant canopy. This pattern limits differentiation among sampling techniques. However, considering the combination of pest density and lower CV values, tray beating proved more effective for this group, although the optimal sampling unit still depends on species–crop interactions.

In contrast, whiteflies showed strong effects of all main factors and significant interactions, reflecting their sensitivity to sampling method. *Bemisia tabaci* is highly polyphagous and occurs in numerous crops, including those evaluated in this study (Lima et al. 2017; Macedo et al. 2019). The relatively lower coefficients of variation indicate a more uniform distribution compared to other pests, likely related to their high reproductive rate and colonization capacity. Whiteflies also showed a broader vertical distribution across plant strata, especially in kale, making the selection of a single optimal sampling unit more difficult. However, despite variation among crops, the combination of higher densities and lower CV values indicates that direct counting is the most effective sampling method. This may be due to the high mobility of adults, which can escape during leaf shaking and thus be underestimated by tray sampling.

For lace bugs, the significant three-way interaction among technique, stratum, and crop indicates a highly complex distribution pattern, strongly dependent on host plant characteristics. *Corythaica passiflorae*, associated with scarlet eggplant, is commonly related to Solanaceae, whereas *Gargaphia lunulata*, observed on okra, is polyphagous (Silva et al. 2024). The higher densities observed in scarlet eggplant, particularly in the middle stratum, suggest that this crop provides favorable conditions for population buildup. In contrast, the lower densities in okra may reflect less suitable conditions for colonization or greater exposure to environmental stress. Both species showed lower preference for the apical stratum, but the optimal sampling unit depends on specific crop × species interactions. Nevertheless, direct counting was the most effective technique for lace bug sampling.

Overall, sampling technique was more consistent across crops within the same pest group, with direct counting recommended for all groups except thrips. However, for all pest groups, the optimal sampling unit varied according to crop–pest interactions. From a practical perspective, sampling techniques can be standardized by pest group, but additional studies are needed to refine sampling units for sap-sucking pests in major vegetable crops grown in agroforestry systems. This would improve sampling efficiency and support decision-making in IPM programs.

## Conclusion

The efficiency of sampling methods for sap-sucking insects varied according to pest group, crop, and plant stratum, highlighting the importance of considering ecological interactions when defining sampling protocols. No single sampling unit was consistently optimal across all conditions.

Direct counting was generally the most effective technique for aphids, whiteflies, and lace bugs, whereas tray beating performed better for thrips, likely due to differences in insect behavior and mobility.

From a practical standpoint, sampling techniques may be standardized according to pest functional groups, but the definition of the most efficient sampling unit should consider crop-specific interactions. These findings contribute to improving pest monitoring strategies and decision-making in IPM programs under tropical and agroecological conditions.

## Data Availability

The datasets generated and/or analyzed during the current study are available from the corresponding author upon reasonable request.
